# How varying CD4 criteria for treatment initiation was associated with mortality of HIV-patients? A retrospective analysis of electronic health records from Andhra Pradesh, India

**DOI:** 10.7189/jogh.10.010408

**Published:** 2020-06

**Authors:** Ram Bajpai, Himanshu K Chaturvedi, Josip Car

**Affiliations:** 1 School of Primary Community and Social Care, Keele University, Newcastle-Under-Lyme, Staffordshire, UK; 2 National Institute of Medical Statistics, Indian Council of Medical Research, New Delhi, India; 3 Centre for Population Health Sciences, Lee Kong Chian School of Medicine, Nanyang Technological University, Singapore; 4 Global eHealth Unit, Department of Primary Care and Public Health, School of Public Health, Imperial College London, London, UK

## Abstract

**Background:**

HIV treatment and care services were scaled up in 2007 in India with objective to increase HIV-care coverage. CD4 count based criteria was mainly used for treatment initiation with increasing threshold in later years. Therefore, this paper aimed to evaluate the survival by varying CD4 criteria for antiretroviral treatment (ART) initiation among of HIV-positive patients, and independent factors associated with the mortality.

**Methods:**

This retrospective cohort study included 127 949 HIV-positive patients aged ≥15 years, who initiated ART between 2007 and 2013 in Andhra Pradesh state, India. The patient’s demographic and clinical characteristics were extracted from the patient’s health records from electronic Computerized Management Information System Software (CMIS). Incidence of mortality/100 person-years was calculated for CD4 and treatment initiation categories. Kaplan-Meier and multivariable Cox-regression analyses were used to explore the association.

**Results:**

Median CD4 count was 172 (inter-quartile range (IQR) = 102-240) at the time of treatment initiation, and 19.3% of them had ≤ 100 CD4 count. Incidence of mortality for the period 2007-08 (CD4 ≤ 200 cells/mm^3^) was 8.5/100 person-years compared to 6.4/100 person-years at risk for the period 2012 onwards (CD4 ≤ 350 cells/mm^3^). Earlier thresholds for treatment initiation showed higher risk of mortality (2007-08 (CD4 ≤ 200 cells/mm^3^), adjusted hazard ratio (HR): 1.86, 95% confidence interval (CI): 1.68-2.07; 2009-11 (CD4 ≤ 250 cells/mm^3^), HR = 1.67, 95% CI = 1.51-1.85) compared to 2012 onwards (CD4 ≤ 350 cells/mm^3^) criteria for treatment initiation.

**Conclusions:**

Increasing CD4 threshold for treatment initiation over time was independently associated with lower risk of mortality. More efforts are required to detect and treat early, monitoring of follow-ups, promote health education to improve ART adherence, and provide supportive environment that encourages HIV-infected patients to disclose their HIV status in confidence.

India formed an AIDS Task Force under the Indian Council of Medical Research (ICMR) to respond HIV/AIDS challenge after the detection of first AIDS case in 1986 [1]. Since then, HIV infections have been reported in all states and union territories. Further, to manage the HIV programme and its implementation, an autonomous National AIDS Control Organisation (NACO) was set up under the ministry of Health [2]. India launched a free antiretroviral treatment (ART) programme in 2004, and more than 400 ART centres are providing HIV care across the nation [3,4].

The ART services were scaled up at national level in 2007 with objective to increase HIV-care coverage. A new treatment criterion (CD4 count ≤ 200 cells/mm^3^ or HIV clinical stage III/IV irrespective of CD4 count, including coinfections such as tuberculosis) for ART initiation was adapted [5]. The CD4 criteria for ART initiation was further modified in 2009 to ≤ 250 cells/mm^3^ and to ≤ 350 cells/mm^3^ in 2012, as recommended by the Joint United Nations Programme on HIV/AIDS (UNAIDS) [6]. Addition to treatment, ART centres also facilitate follow-up monitoring, screening for opportunistic infections, and counselling. The 2013 World Health organisation (WHO) ART guidelines strongly recommended initiating ART for all adults PLHIV with a CD4 count ≤ 500 cells/mm^3^, regardless of WHO clinical stage [7]. Early start of ART is associated with reduced risk of AIDS progression or death, TB, other coinfections and increased likelihood of immune recovery [8-10]. Additionally, evidence from a randomized controlled trial indicated that earlier ART can significantly reduce the risk of HIV to HIV-negative sexual partners in heterosexual couples [11]. The WHO database suggests nearly two-third (60%) of the fifty-eight HIV-focused countries had adopted the CD4 threshold of ≤ 500 cells/mm^3^ for initiating ART, and 7% moved the CD4 threshold to >500 cells/mm^3^ by June 2014 [12]. Although the median CD4 count at the time of ART initiation is progressively increasing, however, it remained significantly lower than 350 cells/mm^3^ in almost all settings [13,14]. Despite the considerable increase in the number of PLHIVs in India accessing ART from 17% in 2007 to 52% in 2013, ART coverage of people eligible for treatment is still low [6,15]. The success of the ART program depends upon proper monitoring for drug adherence, timely presentation and CD4 investigation, as well as motivation for pre-ART cases for on-time visits to the nearest ART centre for their further assessment to prevent the progression and transmission of HIV [6,12-17].

Several single centre-based studies have been conducted in India to look for demographic and clinical factors (such as higher age, male gender, clinical staging III/IV, lesser bodyweight, and low CD4 count etc.) associated with the early mortality [18-21]. However, limited studies have been published at the state level from India to reconfirm these factors. Therefore, the main objective of this study was to explore the survival by varying CD4 criteria of treatment initiation, and the secondary objective was to explore the independent factors associated with the mortality.

## METHODS

### Study Design, period and inclusion

This retrospective analysis included all registered adult (aged ≥ 15 years) HIV/AIDS patients, who initiated ART treatment between January 2007 and December 2013 in undivided Andhra Pradesh state. The patient’s demographic and clinical characteristics were extracted from the patient’s health records from electronic Computerized Management Information System Software (CMIS). We excluded data from the analysis if the patient initiated their treatment elsewhere and registered in Andhra Pradesh, pre-ART cases, ART initiation date was missing, ART started in Andhra Pradesh but later transferred out to other states, and if the status was unknown at the end of the study period.

### State profile

Andhra Pradesh (undivided; as it was divided into Andhra Pradesh and Telangana in 2014) is one of the highly populated southern region state in India. According to the national Census 2011, Andhra Pradesh had 84.6 million population, with a total area of 275 045 km^2^ and population density of 308/km^2^, literacy rate of 67% (male: 74.9%; female: 58.7%), and the sex ratio of 993 females per 1000 males. Administratively, Andhra Pradesh was divided into 23 districts that further divided into 1127 revenue divisions. The state has a vast network of public health facilities, including 20 district hospitals, 56 area hospitals, 117 community health centres, 10 specialty hospitals, and 25 civil dispensaries [22].

### The HIV detection, treatment and burden in Andhra Pradesh

As of December 2014, the state had 1869 integrated and counselling testing centres (ICTCs), including prevention of parent-to-child treatment (PPTCT) centres, 286 government recognized blood banks, and 274 sexually transmitted disease (STD) clinics [23]. There were 56 ART and 161 Link ART centres functional in the state providing free first and second line HIV treatments. The state also had 40 community care centres (CCCs) with the primary duty of counselling to HIV-patients and motivating them to enrol to the HIV programme [3,23]. ART centres primarily located at district hospitals and Link ART centres at sub-district level. According to 2012 estimates, Andhra Pradesh had the second highest HIV prevalence (0.75%) in the age group of 15-49 years after Manipur state (1.22%); however, declined from the 2007-08 estimate. It remained one of the six high prevent states in India with the highest number of PLHIVs (n=419 000) accounting for nearly 20% of total HIV/AIDS cases in the country. The estimated cumulative deaths due to AIDS-related illnesses were highest in Andhra Pradesh (approximately 31 000) in 2011 that declined from 46 000 deaths in 2007 [3,23].

### Enrolment of patients to HIV care

Once an HIV-positive individual reached to an ART centre with an ICTC report, s/he were registered in the pre-ART register for HIV care. Furthermore, demographic details and other relevant information were also recorded in the patient treatment record (also called White Card) and a Green Book was issued. Patients were advised to enrol at the nearest ART centre to their current place of residence to ensure proper adherence to treatment. S/he was re-counselled for implications of being HIV-positive, availability of treatment, positive living, nutrition, and positive prevention, etc. The early testing of their spouse/partner and children was encouraged. Recently detected persons were kept under the supervision of a care coordinator to determine whether a HIV-positive person was treated in the same manner as any other patients at the centre, without any discrimination. Furthermore, the patient was referred to a concerned doctor who conduct a detailed medical examination for WHO clinical staging, opportunistic infections (OIs) and other relevant clinical examinations. Based on the clinical findings, the HIV treatment was initiated as per the NACO guidelines described in the Table S1 in the **Online Supplementary Document** [5].

### Follow-ups and primary event

The primary event of interest was the death of the patients. The date of death for all HIV-infected patients who died from all causes during the study period while on antiretroviral treatment was recorded. Patients missing their follow-up visits for more than three months were counted as lost to follow-up, and the date of the last registered follow-up visit was recorded as the date of follow-up loss. ART for HIV-infected patient who were transferred to another ART centre were recorded as transferred-out cases, and their dates of transfer-out were also recorded. Similarly, patients received medical advice was recorded as on medical advice and their dates of medical advice were also recorded [5].

### Study variables

Patients’ baseline demographic, program-related information and clinical characteristics data were collected. Baseline demographic characteristics included, age (15-24 years, 25-34 years, 35-44 years, and >45 years), sex (male/female), education (no formal education and formal education), marital status (single/married), and employment (employed or not employed). Time-period was divided according to the CD4 criteria for treatment initiation (ie, ≤200 cells/mm^3^ in 2007-2008, ≤ 250 cells/mm^3^ in 2009-2011 and ≤ 350 from 2012 onwards). The HIV surveillance program-related information included date of ART initiation, follow-up status, and last date of ART centre visit. Clinical characteristics at the time of ART initiation included bodyweight (< 45 kg, 45-60 kg and > 60 kg), WHO clinical stage (I, II, III, and IV), and CD4 count (<100 cells/mm^3^, 101-200 cells/mm^3^, 201-250 cells/mm^3^, 251-350 cells/mm^3^, 351-500 cells/mm^3^ and >500 cells/mm^3^). We presented missing data as a separate category in each variable and did not impute any value to figure out their characteristics. Imputation was not considered sensible in this case as data were unlikely to be missing at random.

### Statistical analysis

The data were summarised numerically, in the form of percentages for categorical variables. Mean ± standard deviations (SD) or median with interquartile range (IQR) were used to present quantitative data. Mortality density per 100 person-years was calculated for various CD4 categories and ART criteria years. The Kaplan-Meier (KM) method was used to estimate survival after ART initiation, and the log-rank test was used to determine statistical differences between survival rates in subgroups of the variable [24,25]. Univariable and multivariable Cox-regression analytical methods were applied to model predictors of mortality following treatment initiation [26]. The unadjusted and adjusted hazard ratios (HRs) with 95% confidence intervals (CIs) were estimated. We evaluated departure from the proportional hazard assumption for Cox-regression by tests of Schonefild’s residual and graphical inspection of log-log plots, and all the predictor variables satisfied the criterion of being asymptotic [24]. Data were analysed using Stata for Windows (version 14.0, StataCorp, College Station, TX, USA) software. The two-sided *P*-value <0.05 was considered as the level of significance.

### Data access permission and ethical statement

Individual level anonymised PLHIV data access permission was granted by NACO for undivided Andhra Pradesh under a data sharing scheme with national data analysis plan (NDAP) at phase I for policy making in high prevalent states (vide letter No. z-19019/1/2013-NACO(SIMU)). Data were made available to the first author for his PhD research by NACO for secondary data analysis, and it has been approved by the internal ethics committee. Individual consent was taken the affiliated organisations of NACO during the data collection and therefore, no additional consent was required in this study.

## RESULTS

### Patient characteristics

The baseline patient characteristics according to treatment initiation (based on CD4 criterion) of adult HIV-positive patients on ART are summarised in **Table 1**. Overall, 14.4% deaths were recorded during the study period with high proportion within the first year of treatment start (84.8%; 15 566/18,362). Reported lost to follow up was 17.9% during 2007-08 that significantly decreased (*P* < 0.01) to 6.9% in 2012 onwards. The median age was 34 years, 45.9% females, 45.7% had formal education, 59.4% were employed and 68% were married. The median CD4 count was 172 (IQR 102-240) cells/mm^3^ at the time of ART initiation that consistently increased over time (Figure S1 in the **Online Supplementary Document**).

**Table 1:** Demographic and clinical characteristics of HIV positive patients on antiretroviral treatment registered during 2007-11 in undivided Andhra Pradesh, India

Background profile	Included in the analysis (n = 127 949)	Period of treatment criterion for initiation based on CD4 count		
		**2007-08 (n = 39 542)**	**2009-2011 (n = 79 776)**	**2012 onwards (n = 8631)**
**Patient characteristics**				
Age (in years) at ART initiation, median (IQR)	34 (29-40)	33 (28-39)	35 (29-40)	33 (28-40)
Female, (n, %)	58 677 (45.9)	16 929 (42.8)	36 902 (46.3)	4846 (56.1)
Patients who had formal education, (n, %)	58 462 (45.7)	19 528 (49.4)	35 292 (44.2)	3642 (42.2)
Currently working, (n, %)	75 989 (59.4)	28 530 (72.2)	42 898 (53.8)	4561 (65.8)
Body-weight <45 kg at the time of ART initiation, (n, %)	42 327 (33.1)	13 232 (33.5)	26 831 (33.6)	2264 (26.2)
Married/ cohabiting, (n, %)	87 057 (68.0)	27 128 (68.6)	53 752 (67.4)	6177 (71.6)
CD4 cell count (cells/mm3) at the time ART initiation, median (IQR)	172 (102-240)	144 (85-204)	178 (107-242)	278 (200-333)
CD4 count category (cells/mm^3^), (n, %):				
≤100	24 705 (19.3)	98,58 (24.9)	14 340 (18.0)	507 (5.9)
101-200	37 029 (28.9)	13 491 (34.1)	22 213 (27.8)	1325 (15.4)
201-250	17 849 (14.0)	4517 (11.4)	12 301 (15.4)	1031 (11.9)
251-350	12 512 (9.8)	2212 (5.6)	7058 (8.8)	3242 (37.6)
351-500	5189 (4.1)	982 (2.5)	3549 (4.4)	658 (7.6)
501+	4264 (3.3)	701 (1.8)	3067 (3.8)	496 (5.7)
Missing	26 401 (20.6)	7781 (19.7)	17 248 (21.6)	1372 (15.9)
WHO clinical stage at the time of ART initiation, (n, %):				
I	19 293 (15.0)	3613 (9.1)	12 802 (16.0)	2878 (33.3)
II	50 066 (39.1)	12 952 (32.8)	33 702 (42.2)	3412 (39.5)
III	37 173 (29.1)	14 797 (37.4)	21 505 (27.0)	871 (10.1)
IV	3524 (2.8)	1309 (3.3)	2116 (2.7)	99 (1.1)
Missing	17 893 (14.0)	6871 (17.4)	9651 (12.2)	1371 (15.9)
**Program context**				
Number of ART centres	56	31	45	56
Number of districts covered	25	22	23	25
Number of people registered for ART	291 151	103 946	187 205	-*
Recorded adherence (≥95%) to ART, (%)	89.5	90.3	89.3	86.8
Total recorded deaths, (n, %)	18 362 (14.4)	7061 (17.9)	10 902 (13.7)	399 (4.6)
Deaths within 1st year of ART start, (n, %)	15 566 (12.2)	5618 (14.2)	9563 (12.0)	385 (4.5)
Recorded lost to follow up, (n, %)	16 536 (12.9)	6992 (17.7)	8953 (11.2)	591 (6.9)

ART – antiretroviral treatment

*All these HIV-patients were registered between 2007 and 2011.

### Person-years and survival analysis

**Figure 1:**
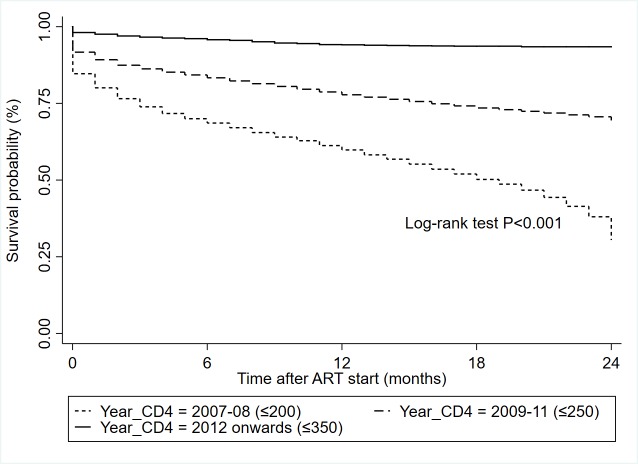
Survival probabilities at 24 months after ART initiation by varying CD4 criteria for treatment initiation.

The study cohort contributed a total of 208 301 person-years at risk during the study period as presented in Table 2. Overall mortality density was 8.8 (95% CI = 8.7-8.9) per 100 person-years at risk. The incidence of mortality was reported higher with the earlier treatment initiation periods (8.5 and 11.0 per 100 person-years in 2007-08 and 2009-11 respectively) compared to 6.4 per 100 person-years from 2012 onwards. The mortality incidence was also consistently decreased when CD4 count increased (13.2/100 person-years for CD4 ≤ 100 to 2.0/100 person-years for CD4 > 500). Overall mean survival during the study period was 66.7 (95% CI = 66.5-67.0) months and significantly different between CD4 subgroups (Figures S2 & S3 in the **Online Supplementary Document**). A twenty-four months survival was recorded to 96.1% who started their treatment with the CD4 count ≤ 350 cells/mm^3^ treatment initiation criteria in 2012 onwards compared to 59.8% survival with CD4 count ≤ 200 cells/mm^3^ criteria in 2007-08 (**Figure 1**).

**Table 2:** Mortality distribution among the HIV-positive patients who had initiated treatment over time with different treatments criteria

Background variables	Overall deaths during study	Deaths within 12 mo (%)	Crude mortality rate (per 100 registrations)	Total person-years at risk	Incidence mortality rates per 100 person-years* (95% CI)
CD4 count (cells/mm^3^) (across the years)					
≤100	5640	4914 (19.9)	22.8	42 657	13.2 (12.8-13.5)
101-200	5117	4222 (11.4)	13.8	62 508	8.2 (7.9-8.4)
201-250	1542	1238 (6.9)	8.6	27 386	5.6 (5.3-5.9)
251-350	804	647 (5.2)	6.4	18 272	4.4 (4.1-4.7)
351-500	204	151 (2.9)	3.9	7504	2.7 (2.3-3.1)
500+	124	93 (2.2)	2.9	6106	2.0 (1.6-2.4)
Missing	4931	4301 (16.3)	18.7	42 539	11.6 (11.2-11.9)
ART criteria year:					
2007-08 (CD4 ≤ 200 cells/mm^3^)	7061	5618 (14.2)	17.9	82 972	8.5 (8.3-8.7)
2009-11 (CD4 ≤ 250 cells/mm^3^)	10 902	9563 (12.0)	13.7	99 467	11.0 (9.9-11.2)
2012 (CD4 ≤ 350 cells/mm^3^)	399	385 (4.5)	4.6	6278	6.4 (5.7-7.0)
Combined	18 362	15 566 (12.2)	14.4	2 871 782	8.8 (8.7-8.9)

ART – antiretroviral treatment, CI – confidence interval

*Calculated as overall deaths divided by total person-years at risk during the study period.

### Risk of mortality with varying CD4 levels over time

The effect of varying CD4 cell count categories on the risk of dying in first year of treatment initiation over time is presented in **Table 3**. Higher rates of mortality (11.3% to 18.6% for 2007-08 period; and 11.7% to 21.1% for 2009-11 period) have been reported compared to 6.9%-11.4% from 2012 onwards when patient level CD4 count ≤ 200 cells/mm^3^. Furthermore, multivariable Cox regression was used to estimate adjusted hazard ratios for 2007-08 (HR range = 1.04-2.36) and (HR range = 0.64-1.73) 2009-11 to explored the trend of risk in different CD4 levels compared to 2012 onwards category. This analysis clearly indicated higher risk of mortality within the first year of treatment start was associated with older treatment initiation criteria (on average 60% higher risk to 2007-08 and 22% higher risk to 2009-11 compared to 2012 onwards).

**Table 3:** Risk of mortality in the first year of treatment initiation with varying CD4 levels in HIV-positive individuals

Patient CD4 levels and year of change in eligibility for treatment	Percent died within first year	Unadjusted HR (95% CI)	Adjusted* HR (95% CI)
CD4 ≤ 100:			
2007-08	18.6	1.47 (1.13-1.90)	1.38 (1.06-1.80)
2009-11	21.1	1.67 (1.29-2.17)	1.66 (1.28-2.15)
2012 (ref)	11.4	1	1
CD4 101-200:			
2007-08	11.3	1.41 (1.14-1.75)	1.34 (1.08-1.65)
2009-11	11.7	1.47 (1.20-1.82)	1.17 (1.17-1.77)
2012 (ref)	6.9	1	1
CD4 201-250:			
2007-08	8.4	1.91 (1.37-2.65)	1.73 (1.24-2.41)
2009-11	6.6	1.52 (1.10-2.30)	1.46 (1.06-2.01)
2012 (ref)	3.8	1	1
CD4 251-350:			
2007-08	8.1	2.51 (1.95-3.24)	2.36 (1.81-3.07)
2009-11	5.4	1.68 (1.33-2.12)	1.61 (1.27-2.03)
2012 (ref)	2.7	1	1
CD4 351-500:			
2007-08	5.3	1.79 (1.02-3.14)	1.72 (0.97-3.04)
2009-11	2.3	0.77 (0.45-1.31)	0.75 (0.44-1.28)
2012 (ref)	2.4	1	1
CD4 501+:			
2007-08	3.1	1.02 (0.51-2.07)	1.04 (0.51-2.12)
2009-11	1.9	0.64 (0.34-1.18)	0.64 (0.35-1.20)
2012 (ref)	2.4	1	1
CD4 – missing:			
2007-08	20.8	3.69 (2.95-4.61)	3.53 (2.81-4.41)
2009-11	15.1	2.46 (1.97-3.06)	2.29 (1.84-2.86)
2012 (ref)	5.9	1	1

Ref – reference category, HR – hazard ratio, CI – confidence interval

*Adjusted for socio-demographic (age, sex, education, marital status, and employment) and clinical (bodyweight and WHO clinical stage) characteristics.

### Factors associated with mortality

The unadjusted and adjusted analysis showed the associated predictors of mortality within the first year of treatment initiation among adult HIV-infected patients in **Table 4**. The risk of mortality within the first-year of treatment initiation was significantly associated with older age (≥45 years, HR = 1.4, 95% CI = 1.27-1.71), male gender (HR = 1.56, 95% CI = 1.51-1.63), with no formal education (HR = 1.16, 95% CI = 1.12-1.20) and working by occupation (HR = 1.17, 95% CI = 1.13-1.21). Further, patients with WHO clinical stage IV had a 1.67-fold increased risk of mortality compared to those at stage I. The likelihood of death among those with a baseline CD4 count of ≤ 100 cells/mm^3^ increased to 9-fold (HR = 9.44, 95% CI = 7.78-11.59), compared to the patients with CD4 count >500 cells/mm^3^.

**Table 4:** Factors associated with mortality after antiretroviral treatment initiation by multivariable Cox regression analysis

Patient characteristics	Within first year of ART initiation		Within the overall study period	
**Unadjusted HR (95% CI)**	**Adjusted HR (95% CI)**	**Unadjusted HR (95% CI)**	**Adjusted HR (95% CI)**
Age (categorical):				
15-24 (ref)	1	1	1	1
25-34	1.28 (1.19-1.37)	1.22 (1.13-1.30)	1.28 (1.21-1.37)	1.22 (1.14-1.30)
35-44	1.55 (1.44-1.66)	1.39 (1.30-1.49)	1.54 (1.44-1.64)	1.38 (1.29-1.47)
45+	1.85 (1.72-1.99)	1.57 (1.46-1.69)	1.85 (1.73-1.98)	1.58 (1.48-1.69)
Sex:				
Male	1.67 (1.61-1.72)	2.05 (1.97-2.13)	1.69 (1.64-1.74)	2.07 (2.00-2.15)
Female (ref)	1	1	1	1
No formal education	1.10 (1.06-1.13)	1.04 (1.01-1.08)	1.10 (1.07-1.13)	1.06 (1.03-1.09)
Formal education (ref)	1	1	1	1
Marital status:				
Single (ref)	1	1	1	1
Married/Live-in	1.02 (0.99-1.06)	0.90 (0.87-0.93)	1.04 (1.00-1.07)	0.90 (0.87-0.93)
Occupation				
Working	1.19 (1.15-1.23)	1.18 (1.14-1.22)	1.18 (1.14-1.22)	1.17 (1.13-1.20)
Not working (ref)	1	1	1	1
Weight (kg):				
<45	3.82 (3.52-4.15)	4.09 (3.76-4.45)	3.48 (3.23-3.74)	3.83 (3.56-4.13)
45-60	1.86 (1.71-2.02)	1.79 (1.64-1.94)	1.80 (1.67-1.93)	1.75 (1.62-1.88)
61+ (ref)	1	1	1	1
missing	3.15 (2.89-3.44)	3.09 (2.82-3.38)	2.95 (2.72-3.19)	2.98 (2.74-3.24)
Treatment start period:				
2007-2008 (≤200 cells/mm^3^)	2.96 (2.67-3.28)	1.68 (1.51-1.87)	3.11 (2.81-3.44)	1.84 (1.66-2.04)
2009-2011 (≤250 cells/mm^3^)	2.45 (2.21-2.72)	1.58 (1.42-1.75)	2.43 (2.20-2.69)	1.61 (1.46-1.79)
2012 onwards (≤350 cells/mm^3^) (ref)	1	1	1	1
CD4 count (cells/mm^3^):				
≤100	10.34 (8.42-12.69)	7.40 (6.02-9.10)	9.01 (7.58-10.82)	6.48 (5.42-7.75)
101-200	5.59 (4.55-6.86)	4.72 (3.84-5.80)	5.11 (4.27-6.10)	4.27 (3.57-5.11)
201-250	3.32 (2.69-4.10)	3.12 (2.52-3.85)	3.13 (2.61-3.76)	2.91 (2.42-3.50)
251-350	2.53 (2.04-3.15)	2.54 (2.04-3.16)	2.44 (2.02-2.95)	2.42 (2.00-2.93)
351-500	1.35 (1.04-1.75)	1.28 (0.99-1.66)	1.37 (1.10-1.72)	1.29 (1.04-1.62)
501+ (ref)	1	1	1	1
missing	8.48 (6.90-10.41)	6.42 (5.23-7.89)	7.48 (6.26-8.94)	5.69 (4.76-6.80)
WHO clinical stage:				
I (ref)	1	1	1	1
II	1.14 (1.08-1.21)	0.96 (0.91-1.02)	1.15 (1.09-1.20)	0.97 (0.92-1.02)
III	1.59 (1.50-1.68)	1.19 (1.13-1.26)	1.55 (1.48-1.63)	1.17 (1.12-1.23)
IV	3.32 (3.06-3.59)	2.02 (1.87-2.19)	3.15 (2.92-3.39)	1.96 (1.82-2.11)
missing	1.44 (1.36-1.54)	0.99 (0.92-1.06)	1.42 (1.34-1.50)	0.97 (0.91-1.04)

ART – antiretroviral treatment, HR – hazard ratio, CI – confidence interval, ref – reference category, WHO – World Health Organisation

## DISCUSSION

The ART scaling-up service has resulted greater coverage of HIV-patients at the early stages. Our findings clearly indicate higher incidence of mortality for earlier treatment initiation criteria – 8.5/100 persons-years for 2007-08 period (CD4 ≤ 200 cells/mm^3^), and 11.0/100 person-years for 2009-11 period (CD4 ≤ 250 cells/mm^3^) – compared to 2012 onwards (CD4 ≤ 350 cells/mm^3^) criteria. Further, the overall excess risk of death was 84% (HR = 1.84, 95% CI = 1.66-2.04) for 2007-08 period (CD4 ≤ 200 cells/mm^3^), and 61% (HR = 1.61, 95% CI = 1.46-1.79) for 2009-11 period (CD4 ≤ 250 cells/mm^3^) compared to 2012 onwards (CD4 ≤ 350 cells/mm^3^) criteria. These findings are in agreement with other studies published from resource-limited settings [9,27,28]. An increased level of CD4 count is always linked with a decreased risk of a new AIDS event or death [29-31]. Furthermore, a multinational study showed that early start of ART could increase life expectancy of HIV-positive persons to the general population [29-32].

Decentralisation of HIV care delivery has increased population coverage and encouraged early access to ART centres with lower travel costs compared to hospitals [29,30]. Patients who approach ART centre at their early stage could delay complex disease conditions due to convenient and easier access to HIV care facility. Therefore, decentralisation of ART services seems to be associated with earlier presentation to HIV care, which might provide opportunity to many patients to initiate early treatment during the disease progression. Increasing numbers of patients have been shown an inclination to start ART in the early stages [4,6].

This retrospective analysis showed 85% of the total deaths have been occurred within the first year of treatment start. This indicates majority of these patients were accessed ART at the later stage of their disease, and explained by the fact that more than one-third (38.4%) of these patients had both CD4 cell count ≤ 200 cells/mm^3^ and WHO clinical stage III/IV at the time of treatment start. Other Indian [9,16,18,19] and international [10,33-36] studies conducted in the other low- and middle-income countries confirmed similar findings. Several self-perceived personal (such as service delivery, financial, personal health perception, and logistical) and structural barriers may also play a significant role in delaying ART accessing at the earlier stage [37].

The CD4 count is an established indicator of the disease severity that directly related to the functional and immune status of a patient, and risk of early mortality among those with lower CD4 counts [38]. Our analysis also showed higher risk of mortality with CD4 count ≤ 350 cells/mm^3^. A study in Hong Kong showed 2.69-fold risk of death when they start treatment with a CD4 count <100 cells/mm^3^ during the follow-up period [39]. Additionally, a study in South Korea showed that patients with CD4 cell count <200 cells/mm^3^ had about 5-fold higher mortality than did those with ≥ 500 cells/mm^3^ [35]. The CD4 cell count is one of the key predictor of mortality, as confirmed by several other studies [35,36,39] but some of studies did not show any association with mortality [10,40]. This may be partly explained by the way CD4 count measured (continuous or categorical), type of categorisation used, and number of events observed in different CD4 count subgroups, that could have made it statistically unreliable.

Males were more at risk for HIV-infection who probably acquired it from the high-risk population (such as female sex workers) and transmit to their female partner(s). Our findings are similar with other studies from under-developed nations [41,42], but not all of them [34,43]. Females are regularly tested during the antenatal care visits or when their spouse show any symptoms leading to earlier diagnosis and treatment of HIV, which could be a reason for their decreased risk of mortality. Though, a gender-wise separate analysis in the same state indicated an increased risk of death in older females with the advanced clinical stages and lower CD4 counts [9]. This could be a result of social discrimination against females, awareness regarding HIV, and no direct health care facilities. Similar pattern was also observed in some previous studies from resource-limited settings [21,40]. These, differences may vary with geography and social structure of the society. Our analysis showed six years of mean survival after initiation of the antiretroviral treatment, which is alike with other studies [21,43], although it remains lower than the developed nations [8,39]. Possible explanations include a lack of awareness, accessibility of the treatment, and social stigma.

The leading causes of HIV-related deaths are mainly due to immune reconstitution syndrome and opportunistic infections. According to a recent report, patients who start their HIV-treatment at late clinical stages (such as III/IV) are at an increased risk of dying [6]. Late presentation to HIV care could also be related to self-perceived barriers such as service delivery, finance, personal health perception etc. A study from South Africa showed forty-four percent participants had at least one barrier and twenty-four percent reported more than three barriers to care that increases excess mortality from fifty percent to eighty percent [37]. Late presentation, either at advanced stages of the disease or with very low CD4 counts, is alarmingly high in both developing and developed countries [10,21,44]. Delayed testing and presentation at HIV care centres is a consequence of the appearance of symptoms, while earlier diagnosis and presentation is the result of self-perceived risk or universal screening. Early disease stage presentation at ART centres will benefit to prevent the progression of AIDS complexity and increasing survival.

### Limitations

There were several limitations in this study, and results may carefully be interpreted in the light of these limitations. The electronic database did not have information on causes of death, details of treatment regimen, incomplete data in several important variables, and opportunistic infections. Although these details were routinely entered in the ART registers. Several important variables such as haemoglobin, height and other clinical information were dropped from the analysis due to incomplete information in the electronic database. Additionally, lost to follow-up cases were intractable for outcome assessment and may have been at increased risk of dying. This study was retrospectively conducted; therefore, limitations of retrospective studies were also included in this study.

## CONCLUSIONS AND IMPLICATIONS

In conclusion, this study compared the effect varying CD4 criteria for treatment initiation with mortality over time in Andhra Pradesh, India. Increased risk of mortality was found to be associated with the earlier CD4 cut-offs (≤ 200 cells/mm^3^ in 200708, and ≤ 250 cells/mm^3^ in 2009-11) for treatment initiation compared to ≤ 350 cells/mm^3^ from 2012 onwards criteria. It indicates the requirement of more efficient and inclusive HIV programme to respond to the HIV epidemic in the state and country at large. As majority of the HIV-patients were died within the first year of treatment initiation, indicating more efforts are required to detect early, emphasis on regular follow-ups, promote health education to improve ART adherence, and provide supportive environment that encourages HIV-infected patients to disclose their HIV status in confidence.

## Additional material

Online Supplementary Document
