# Supplementary material for: How varying CD4 criteria for treatment initiation was associated with mortality of HIV-patients? A retrospective analysis of electronic health records from Andhra Pradesh, India
Source: J Glob Health. 2020 Mar 30;10(1):010408. doi: 10.7189/jogh.10.010408 (PMC7125424; doi:10.7189/jogh.10.010408)

Table S1: Initiation of antiretroviral treatment according to WHO clinical staging and CD4 counts at various times in India (Source: NACO)$

Category of patients	Treatment criteria according to years		
	**2007–2008**	**2009–2011**	**2012 onwards**
**1. For HIV infected adult/ adolescents (including pregnant women)**			
(a) Clinic Stage I and II	Treat if CD4 ≤ 200 cells/ml	Treat if CD4 ≤ 250 cells/ml	Start ART if CD4 ≤ 350 cells/ml
(b) Clinical Stage III and IV	1. Treat if CD4 count ≤ 350 for stage III patients.	1. Treat if CD4 count ≤ 350 for stage III patients.	Start ART irrespective of CD4 count
	2. Treat irrespective of CD4 count for stage IV patients	2. Treat irrespective of CD4 count for stage IV patients	
**2. For HIV and tuberculosis (TB) co-infected patients**			
Patients with HIV and TB co-infection (Pulmonary/Extra Pulmonary)	**Pulmonary –** start ART within 2 weeks of initiation of ATT from all patients with CD4 ≤ 350 cells/ml (for patients with CD4 more than 350, defer ART). **Extra Pulmonary –** start ART within 2 weeks of initiation of ATT in all patients, irrespective of the CD4 count.	**Pulmonary –** start ART within 2 weeks of initiation of ATT from all patients with CD4 ≤ 350 cells/ml (for patients with CD4 more than 350, defer ART). **Extra Pulmonary –** start ART within 2 weeks of initiation of ATT in all patients, irrespective of the CD4 count.	Start ART irrespective of CD4 count and type of tuberculosis (Start ATT first, initiate ART as early as possible between 2 weeks to 2 months when TB treatment is tolerated)
**3. For HIV and Hepatitis B and C co-infected patients**			
**(a)** HIV and HBV/HCV co-infected patients without any evidence of chronic active Hepatitis	Start ART if CD4 ≤ 200 cells/ml	Start ART if CD4 ≤ 250 cells/ml	Start ART if CD4 ≤ 350 cells/ml
**(b)** HIV and HBV/HCV co-infected patients with documented evidence of chronic active hepatitis	Start ART irrespective of the CD4 count	Start ART irrespective of the CD4 count	Start ART irrespective of the CD4 count
**Specific situations:-**			
a. HIV and tuberculosis (start Efavirenz-based regimen)			
b. Special attention to monitor hepatotoxicity			
c. HIV and pregnancy (avoid Efavirenz in the first trimester)			
d. For WHO stage I and II patients - repeat CD4 counts after four weeks			
e. WHO clinical stage III and IV patients will be strictly monitored for adverse effects of nevirapine			
**In the absence of a CD4 count, do not delay ART initiation if the patient is clinically eligible according to the WHO Clinical Staging Criteria**			

$NACO. National Guidelines for Antiretroviral Therapy. New Delhi: National AIDS Control Organization (NACO), Ministry of Health & Family Welfare, India, 2007-2012.

Figure S1: Distribution of median CD4 cell count according to calendar year of registration at different ART centres in Andhra Pradesh, India

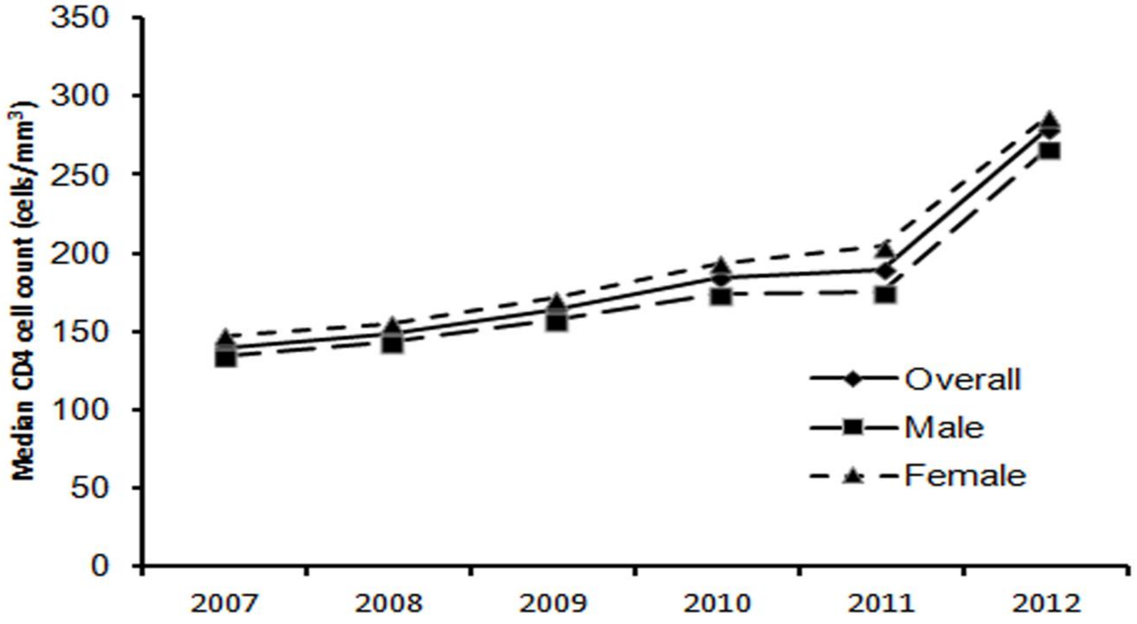

Figure S2: Overall survival curve during the study period

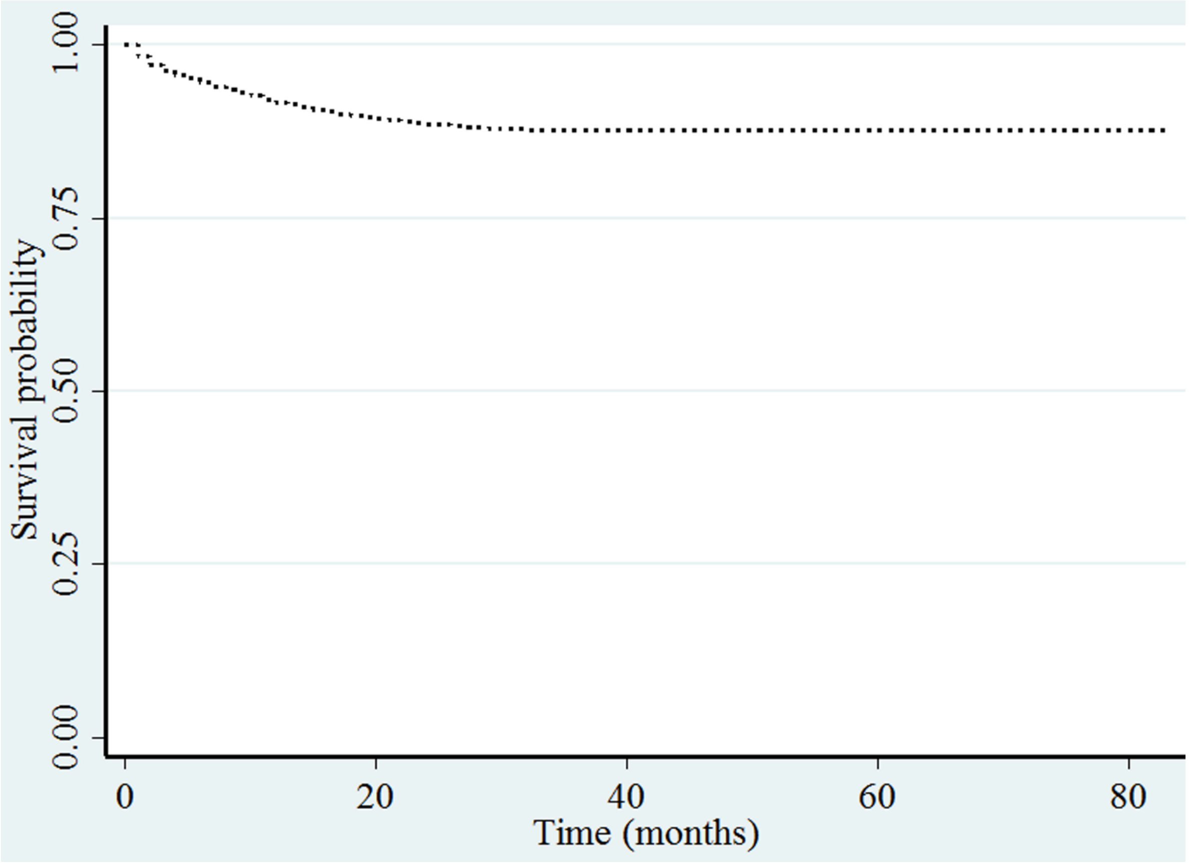

Figure S3: Overall survival probabilities according to the CD4 subgroups

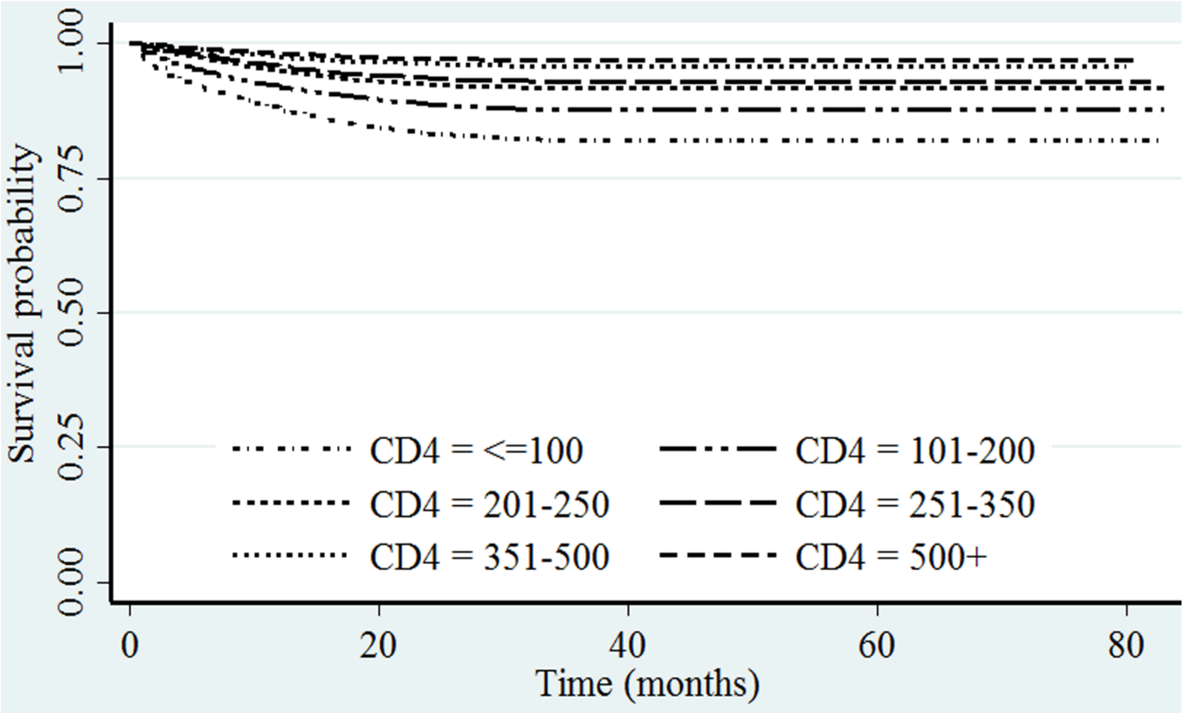

Supplement: Online Supplementary Document [file jogh-10-010408-s001.pdf]
